# Pseudoknots: RNA Structures with Diverse Functions

**DOI:** 10.1371/journal.pbio.0030213

**Published:** 2005-06-14

**Authors:** David W Staple, Samuel E Butcher

## Abstract

Just as proteins form distinct structural motifs, certain structures are commonly adopted by RNA molecules. Amongst the most prevalent is the RNA pseudoknot.

RNA molecules fulfill a diverse set of biological functions within cells, from the transfer of genetic information from DNA to protein, to enzymatic catalysis. Reflecting this range of roles, simple linear strings of RNA—made up of uracil, guanine, cytosine, and adenine—form a variety of complex three-dimensional structures. Just as proteins form distinct structural motifs such as zinc fingers and beta barrels, certain structures are also commonly adopted by RNA molecules. Among the most prevalent RNA structures is a motif known as the pseudoknot. First recognized in the turnip yellow mosaic virus [1], a pseudoknot is an RNA structure that is minimally composed of two helical segments connected by single-stranded regions or loops (Figure 1). Although several distinct folding topologies of pseudoknots exist, the best characterized is the H type. In the H-type fold, the bases in the loop of a hairpin form intramolecular pairs with bases outside of the stem (Figure 1A and 1B). This causes the formation of a second stem and loop, resulting in a pseudoknot with two stems and two loops (Figure 1C). The two stems are able to stack on top of each other to form a quasi-continuous helix with one continuous and one discontinuous strand. The single-stranded loop regions often interact with the adjacent stems (loop 1–stem 2 or loop 2–stem 1) to form hydrogen bonds and to participate in the overall structure of the molecule. Hence, this relatively simple fold can yield very complex and stable RNA structures. Due to variation of the lengths of the loops and stems, as well as the types of interactions between them, pseudoknots represent a structurally diverse group. It is fitting that they play a variety of diverse roles in biology. These roles include forming the catalytic core of various ribozymes [2,3], self-splicing introns [4], and telomerase [5]. Additionally, pseudoknots play critical roles in altering gene expression by inducing ribosomal frameshifting in many viruses [6–9].

**Figure 1:**
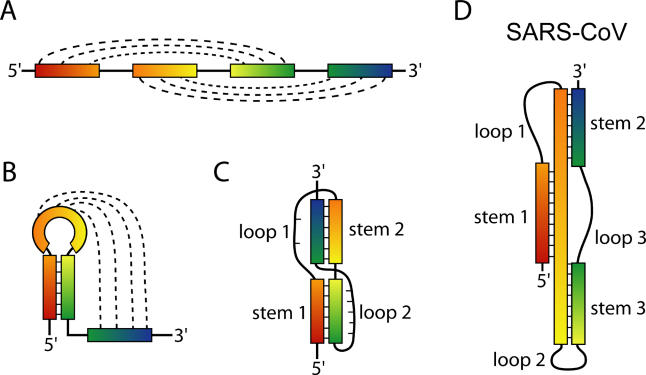
RNA Pseudoknot Architecture (A) Linear arrangement of base-pairing elements within an H-type RNA pseudoknot. Base pairing is indicated with dashed lines. (B) Formation of initial hairpin within pseudoknot sequence. Base pairings from loop to bases outside the hairpin are indicated with dashed lines. (C) Classic H-type pseudoknot fold. (D) Three-stemmed RNA pseudoknot fold from SARS-CoV.

## Catalytically Active Pseudoknots

**Figure 2:**
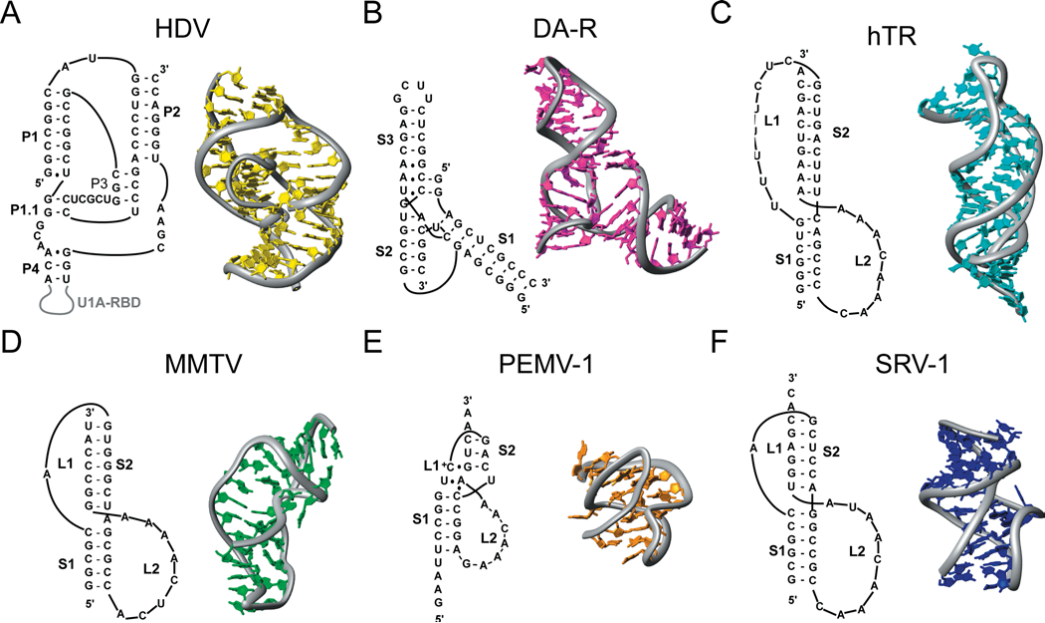
Sequences and Structures of RNA Pseudoknots Stems and loops are numbered sequentially, unless otherwise noted. Structure coordinates were obtained from the Protein Data Bank (http://www.rcsb.org), and structural representations were produced using MOLMOL software. (A) HDV (1SJ3). Numbering of stems reflects standard nomenclature for HDV. The U1A RNA binding domain is colored gray and is not included in the three-dimensional structure. (B) Diels-Alder ribozyme (DA-R) (1YLS). (C) Human telomerase (hTR) (1YMO). (D) MMTV (1RNK). (E) Pea enation mosaic virus RNA1 (PEMV-1) (1KPZ). (F) Simian retrovirus 1 (SRV-1) (1E95).

Hepatitis delta virus (HDV) is a satellite virus of hepatitis B virus. Infection of humans by both HDV and hepatitis B virus is generally more severe than a hepatitis B virus infection alone [10]. HDV has a circular genome that is replicated by the host RNA polymerase II through a double-rolling-circle mechanism. This mechanism produces long strands of RNA that must be processed into unit lengths for viral replication. The processing of the viral RNA is achieved by the self-cleaving HDV ribozyme encoded in the RNA [11]. The HDV ribozyme folds into a double-pseudoknot conformation and self-cleaves, producing single-genome-length HDV RNAs. The HDV ribozyme is the fastest-known naturally occurring self-cleaving ribozyme, with a cleavage rate greater than one per second, and is active in vitro in the absence of any proteins [12]. The HDV ribozyme consists of five helical segments that form two coaxial stacks of two (stems P2 and P3) and three (stems P1, P1.1, and P4) helices each (Figure 2A) [3,13]. Two pseudoknots are formed, each with one helix from each coaxial stack (stems P1 and P2, and stems P3 and P1.1). These two pseudoknots stack on top of each other, forming a nested double-pseudoknot conformation [13].

The removal of introns from pre–messenger RNA (pre-mRNA) is fundamentally important for eukaryotic life. Most introns are removed by a ribonucleoprotein complex called the spliceosome. A subset of introns are self-cleaving, catalyzing their own removal from pre-mRNA without the aid of proteins [14]. One such class of introns are the group I self-splicing introns, with the most well-studied example being from the ciliate *Tetrahymena*. The structure of this ribozyme is made up of three helical domains, with many tertiary contacts between the domains [15]. The only portion of the RNA that spans all three helical domains is a pseudoknot belt that wraps around the molecule, base-pairing with all three helices [15]. The pseudoknot establishes the catalytic core of the group I self-splicing introns.

Naturally occurring ribozymes appear to perform mainly hydrolysis and transesterification reactions [16]; however, in vitro selection has yielded RNAs capable of performing a wide variety of enzymatic reactions [17]. Recently the structure of an RNA capable of catalyzing carbon–carbon bond formation by the Diels-Alder reaction was solved (Figure 2B) [18]. The RNA adopts a λ-shaped fold of its three helices in which stems 2 and 3 stack coaxially, with stem 1 abutting the active site, forming a pocket precisely complementary to the reaction product. The 5′ end of the RNA bridges helical stems 3 and 1, generating a complex nested pseudoknot topology. Although conformationally distinct from the HDV ribozyme [3], it is worthwhile to note that they are two of the fastest-known ribozymes, and both utilize a nested pseudoknot architecture [18].

Chromosomes possess protective ends known as telomeres to protect themselves from degradation due to successive rounds of DNA synthesis. Telomerase, the ribonucleoprotein complex responsible for the maintenance of the telomere ends [19], is upregulated in most cancers [20] and might play a role in aging [21]. Human telomerase is made up of a 451-nucleotide RNA, a reverse transcriptase, and other proteins [22]. At the 5′ end of the RNA is a highly conserved pseudoknot, required for activity, which lies at the core of telomerase. The structure of the human telomerase pseudoknot reveals a classic H-type pseudoknot fold with a slight bend between the stems (Figure 2C) [5]. A triple-helix structure flanks the junction of the helices and extends into each stem. Mutations within the telomerase pseudoknot have been directly linked to the diseases autosomal dyskeratosis congenita [21] and aplastic anemia [23].

## Frameshift-Inducing Pseudoknots

Not all pseudoknots with biological functions are catalytically active. In fact, one of the most common functions of pseudoknots is to induce ribosomes to slip into alternative reading frames, otherwise known as frameshifting. Ribosomes typically translate mRNA without shifting the translational reading frame [24]. However, a number of organisms have evolved mechanisms to cause site-specific or programmed frameshifting of the ribosome in either the +1 or −1 direction [25]. Programmed −1 ribosomal frameshifting is typically found in viruses and is required for the replication and proliferation of all retroviruses. Therefore, the pseudoknot structures involved in frameshifting are attractive targets for the development of antiviral drugs. The frameshift event is induced by two RNA elements within the mRNA: (i) a heptanucleotide slippery sequence X XXY YYZ (spaced triplets represent preframeshift codons) and (ii) a downstream RNA structure, typically a pseudoknot [26]. The mechanism behind how these elements promote −1 frameshifting is not fully understood. The current model posits that the ribosome encounters the downstream pseudoknot while the slippery sequence is being decoded by the ribosome. The pseudoknot structure likely causes the ribosome to pause, which is necessary but not sufficient for frameshifting to occur [27]. While paused on the slippery sequence, the ribosome slips back one nucleotide and subsequently continues translation in the −1 reading frame.

The nuclear magnetic resonance (NMR) structure of the mouse mammary tumor virus (MMTV) frameshift-inducing pseudoknot was the first structure of a frameshift-inducing pseudoknot (Figure 2D) [6]. The MMTV pseudoknot forms a compact structure of two guanine/cytosine-rich A-form helices. The MMTV pseudoknot has a bend of approximately 60° between the two helices, caused by an unpaired adenine that intercalates between the helices and may act as a hinge. Subsequent structural and functional studies of several variants of the MMTV pseudoknot reveal that the intercalated nucleotide and the resulting bend between stems 1 and 2 are required for efficient frameshifting [28].

In beet western yellow virus, pea enation mosaic virus, and other luteoviruses, an RNA pseudoknot also stimulates a −1 frameshift between the *P1* and *P2* genes [29]. These structures, solved by X-ray crystallography and NMR, respectively, revealed compact H-type pseudoknots with extensive loop–stem interactions (Figure 2E) [7,9]. Like that of MMTV, frameshift-inducing pseudoknots in both the beet western yellow virus and pea enation mosaic virus have an unpaired nucleotide at the junction of the stems; however, this nucleotide is displaced from the helix, not intercalated as in MMTV.

The frameshift-inducing pseudoknot from simian retrovirus 1 contains a number of unique features (Figure 2F) [8]. Although predicted to resemble that of MMTV, with an unpaired adenine between the helices, the structure revealed the formation of a uracil–adenine pair at the junction, allowing the two stems to stack directly on top of each other (Figure 2F) [8]. The simian retrovirus 1 pseudoknot forms an extensive loop 2–stem 1 triplex, which contains a ribose zipper motif in addition to base–base and base–sugar interactions [8].

The severe acute respiratory syndrome coronavirus (SARS-CoV) genome contains two large genes, *ORF 1a* and *ORF 1b*, separated by a programmed −1 frameshift element required for *ORF 1b* expression [30]. Recent work has suggested that the SARS-CoV frameshift-inducing pseudoknot may be unique because it contains a third stem–loop [31,32]. In this issue of *PLoS Biology*, bioinformatic, phylogenetic, and structural evidence is reported indicating that the SARS-CoV pseudoknot is indeed a three-stemmed RNA pseudoknot (see Figure 1D) [33]. Dinman and co-workers report the potential for the formation of this three-stemmed pseudoknot in all coronaviruses in the GenBank database. NMR experiments confirmed the proposed three-stemmed pseudoknot structure in SARS-CoV. Although the atomic-resolution structure has not yet been determined, this study identifies a new secondary structure capable of promoting frameshifting that is structurally distinct from previously described pseudoknots (see Figure 1D).

RNA pseudoknots have been identified in nearly every organism and comprise functional domains within ribozymes, self-splicing introns, ribonucleoprotein complexes, viral genomes, and many other biological systems. It is clear that the pseudoknot topology can result in many different, complex structures. The pseudoknot, therefore, represents an important piece of RNA architecture, as it provides a means for a single RNA strand to fold upon itself to produce a globular structure capable of performing important biological functions.

## Abbreviations

HDV: hepatitis delta virus
MMTV: mouse mammary tumor virus
mRNA: messenger RNA
NMR: nuclear magnetic resonance
SARS-CoV: severe acute respiratory syndrome coronavirus
